# Transferred PMN-PT Thick Film on Conductive Silver Epoxy

**DOI:** 10.3390/ma11091621

**Published:** 2018-09-05

**Authors:** Tao Zhang, Jun Ou-Yang, Xiaofei Yang, Benpeng Zhu

**Affiliations:** 1 School of Optical and Electronic Information, Huazhong University of Science and Technology, Wuhan 430074, China; zhangtao@163.com (T.Z.); ouyanjun@163.com (J.O.-Y.); yangxiaofei@163.com (X.Y.); 2 State Key Laboratory of Transducer Technology, Chinese Academy of Sciences, Shanghai 200050, China

**Keywords:** PMN-PT, piezoelectric thick film, transfer, conductive epoxy

## Abstract

Approximately 25 μm Pb(Mg_1/3_Nb_2/3_)O_3_–PbTiO_3_ (PMN-PT) thick film was synthesized based on a sol-gel/composite route. The obtained PMN-PT thick film was successfully transferred from the Silicon substrate to the conductive silver epoxy using a novel wet chemical method. The mechanism of this damage free transfer was explored and analyzed. Compared with the film on Silicon substrate, the transferred one exhibited superior dielectric, ferroelectric and piezoelectric properties. These promising results indicate that transferred PMN-PT thick film possesses the capability for piezoelectric device application, especially for ultrasound transducer fabrication. Most importantly, this chemical route opens a new path for transfer of thick film.

## 1. Introduction

Ultrasound, one of the most important tools for biomedicine, has been intensively investigated for medical imaging [1], therapy [2] and manipulation [3]. To meet the requirements of precise medicine, ultrasound beams need to be much narrower. This will benefit high resolution medical applications. Current trends indicate that the operational frequency of the ultrasound transducer is greater than 50 MHz [4,5,6,7,8,9]. The piezoelectric layer is the core part of the ultrasound transducer. Its thickness needs to be inversely proportional to the working frequency. To produce a high frequency (>50 MHz) ultrasound, the thickness of the piezoelectric layer is usually less than 40 m. Traditional lapping down technology for bulk material is no longer useful. Therefore, the development of piezoelectric thick film offers a promising solution [10].

Among piezoelectric thick films, Pb(Mg_1/3_Nb_2/3_)O_3_–PbTiO_3_ (PMN-PT) thick film, especially with a composition near the morphotropic phase boundary, is famous for its desirable dielectric constant and its excellent piezoelectric performance. This indicates that this kind of piezoelectric thick film could be useful for the fabrication of ultrasound transducers with an operation frequency greater than 50 MHz [11,12,13]. When building a transducer, a backing layer is indispensable because it can reduce ring-down time and back reflections. E-solder 3022, a conductive silver epoxy, is commonly used as a backing layer. This conductive backing layer can improve the device’s electrical connection. In traditional transducer fabrication processes, E-solder 3022 needs to be deposited onto the piezoelectric layer after the bulk material is lapped down to the desired thickness. Today, piezoelectric thick film is usually prepared on the Si substrate and cannot be directly deposited onto the E-solder 3022 backing layer. Therefore, it is of great significance to investigate the transfer of PMN-PT thick film from Silicon substrate onto conductive silver epoxy.

In this study, approximately 25 m PMN-PT thick film with the nominal composition of 0.65Pb(Mg_1/3_Nb_2/3_)O_3_-0.35PbTiO_3_ was prepared on a Pt(111)/Ti/SiO_2_/Si(100) substrate using a sol-gel/composite. A simple chemical transfer route was introduced and its mechanism was explored and analyzed. The electrical performance of the PMN-PT thick film was identified before and after transfer.

## 2. Materials and Methods

To prepare the PMN-PT precursor, lead acetate trihydrate (Pb(C_2_H_3_O_2_)∙3H_2_O,), magnesium ethoxide (Mg(OC_2_H_5_)_2_), niobium ethoxide (Nb(OC_2_H_5_)_5_), and titanium isopropoxide (Ti(OC_3_H_7_)_4_) were selected as starting materials, and 2-methoxyethanol was employed as the solvent [13]. To get the PMN-PT composite solution, the obtained sol-gel solution and PMN-PT powder (Piezo Specialties, Ltd., Guangzhou, China) were mixed with a mass ratio of 4:1, and were ball-milled (Planetary ball mill PM 400, Retsch, Haan, Germany) for 24 h. Using spinning coating technology (Spin Coater KW-4, Chemat Technology, Northridge, CA, USA), the PMN-PT sol-gel and PMN-PT powder composite was deposited onto the platinum-buffered Silicon substrate (Pt(111)/Ti/SiO_2_/Si(100)) at a speed of 3000 rpm for 30 s. Two pyrolysis operations (Hotplate, Brewer Science, Rolla, MO, USA) were carried out for each layer: 1-min heat treatment at 250 °C to drive out the solvent, and another at 400 °C for 1 min to decompose organic compounds. Then, based on rapid thermal annealing technology, each layer was subjected to a sintering process at 650 °C for 60 s. In order to obtain the desired thickness, this step was repeated layer by layer. Finally, the sample was sintered in the furnace under air atmosphere at 750 °C for 1 h.

To realize the PMN-PT thick film’s transfer, a gold layer (100 nm) was sputtered (Sputtering K550X, Emitech Ltd., Ashford, UK) onto the film’s surface. E-solder 3022 was deposited onto the Au layer. After the conductive silver epoxy was centrifuged and cured, the backing layer was ground to approximately 2 mm. Then, the sample was diced into a regular sized stack. The most important step was to place the obtained stack into the 20% KOH solution at 80 °C. About 5 min later, the PMN-PT thick film with E-solder 3022 separated from the Pt(111)/Ti/SiO_2_/Si(100) substrate.

## 3. Results and Discussion

**Figure 1:**
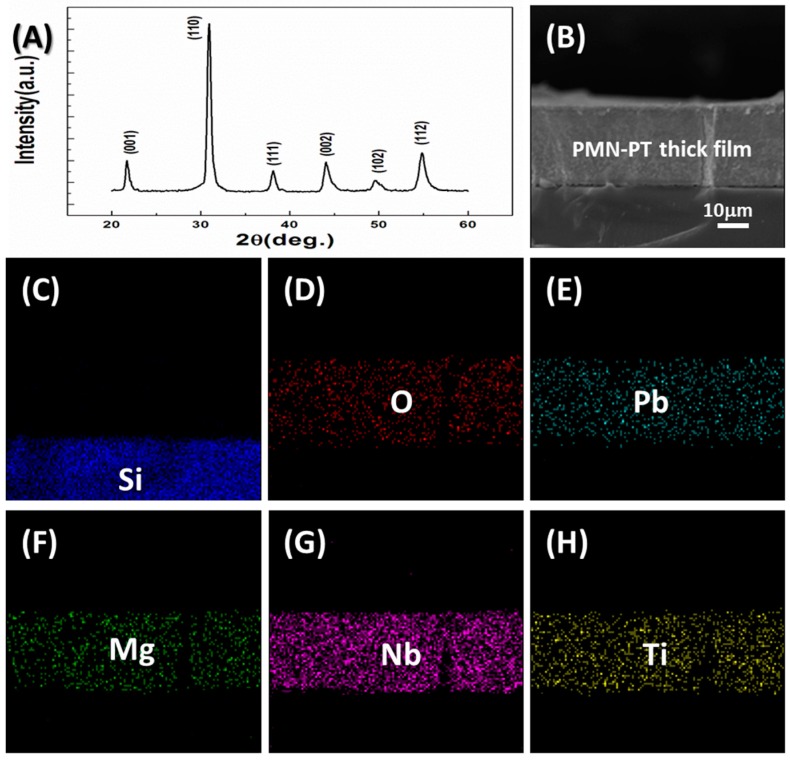
(**A**) XRD pattern and (**B**) SEM cross-sectional image of Pb(Mg_1/3_Nb_2/3_)O_3_–PbTiO_3_ (PMN-PT) thick film on silicon wafer; (**C**–**H**) cross-sectional elements distribution in the PMN-PT thick film on silicon substrate.

The film’s X-ray diffraction (XRD, XRD-7000, Shimadzu Coprporation, Kyoto, Japan) pattern is shown in Figure 1A. It is clear that the obtained PMN-PT thick film is in a well-crystallized perovskite structure. No pyrochlore second phase can be detected. As shown in Figure 1B, the PMN-PT thick film on the Si wafer is highly dense and crack-free. Its porosity is around 96.7% and its thickness is approximately 25 μm. In Figure 1C–H, it is easy to see that the substrate is a silicon wafer. The elements O, Pb, Mg, Nb and Ti are distributed in the film, indicating that the obtained thick film is indeed PMN-PT.

**Figure 2:**
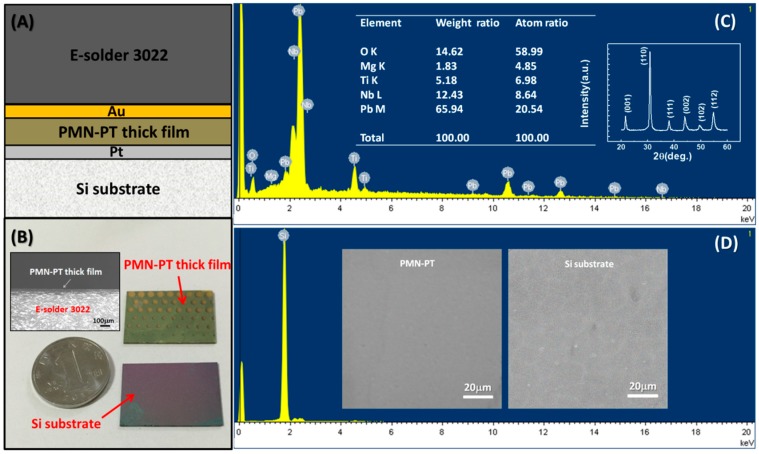
(**A**) The schematic diagram of PMN-PT thick film on platinum-buffered Si substrate after E-solder 3022 deposition; (**B**) photograph of PMN-PT thick film and Si substrate after transfer process, the inset: SEM morphology of PMN-PT thick film on E-solder 3022; EDS analysis results of newborn surfaces of (**C**) PMN-PT thick film and (**D**) Si substrate. The inset: XRD pattern of PMN-PT film after transfer; SEM surface image of PMN-PT film and Si substrate.

Figure 2A describes the stack structure of PMN-PT thick film on platinum-buffered Silicon substrate after E-solder 3022 deposition. After 5 min of being immersed in the 20% KOH solution at 80 °C, the PMN-PT thick film with E-solder 3022 would separate from the Si substrate, as shown in Figure 2B. In other words, the thick film was transferred from Si substrate to conductive silver epoxy successfully. In order to explore the mechanism of the PMN-PT thick film’s transfer, we investigated the newborn surfaces of the PMN-PT film and the Si substrate. The XRD pattern for the PMN-PT film after transfer is illustrated in the inset of Figure 2C. Compared with Figure 1A, no obvious difference can be found. The energy-dispersive X-ray spectroscopy (EDS, Hitachi, Tokyo, Japan) results of the film and substrate are depicted in Figure 2C,D, respectively. In the film, there are five elements: Pb, Mg, Nb, Ti and O, with the atom ratio of 20.54:4.85:8.64:6.98:58.99. This atom ratio is similar to the chemical formula 0.65Pb(Mg_1/3_Nb_2/3_)O_3_-0.35PbTiO_3_. However, this substrate is only comprised of Si. The Pt element was not detected in the EDS test. In the experiment, we noticed that the Pt electrode was attached to the PMN-PT thick film after transfer the process. When we used a cotton swab to touch it slightly, the Pt electrode would come off. This indicates that the Pt electrode acts as a sacrificial layer in the PMN-PT thick film transfer process. The Scanning Electron Microscope (SEM, S-3500N, Hitachi, Tokyo, Japan) micrographs of the PMN-PT thick film and Silicon substrate are presented in the inset of Figure 2D. Both of the newborn surfaces are smooth and no obvious crack can be found. This indicates that this transfer route causes no physical damage to the PMN-PT film.

**Figure 3:**
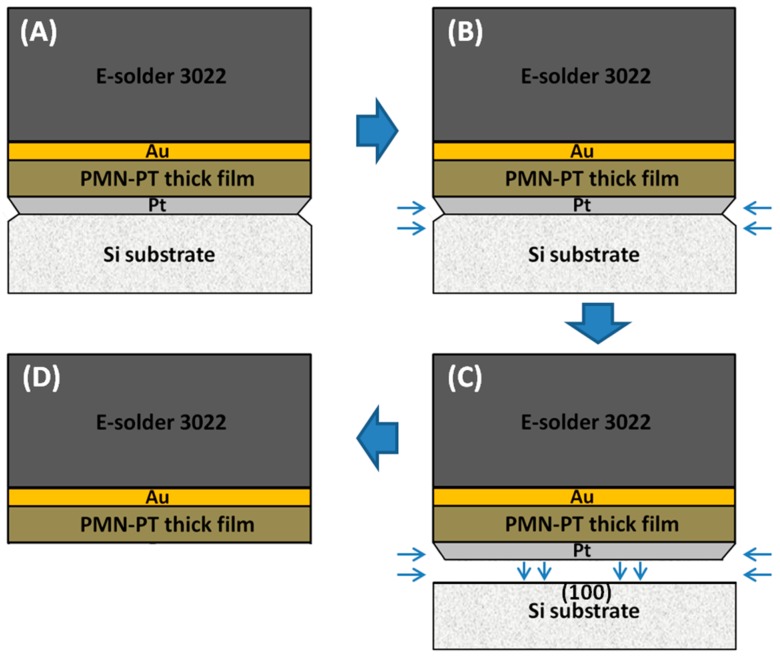
The schematic diagram of PMN-PT thick film transfer process: (**A**) after dicing; (**B**) etching; (**C**) after etching; (**D**) after transfer.

The mechanism of the PMN-PT film’s transfer is demonstrated in Figure 3. Due to the fragility of the silicon, some micro newborn silicon surface appeared at the interface of the silicon substrate and the Pt electrode in the dicing process, as shown in Figure 3A. When the stack was immersed in the alkaline solution, the chemical corrosion would happen at those newborn places immediately. The silicon used here is monocrystalline with (100) crystallographic orientation. Compared with other silicon planes, the (100) silicon plane has the fastest etching speed in alkaline solution [14]. This is because corrosion occurs along the Si (100) plane from all edges simultaneously, as presented in Figure 3C. Within five minutes, the transfer of PMN-PT thick film from silicon substrate to conductive silver epoxy was successfully realized.

**Figure 4:**
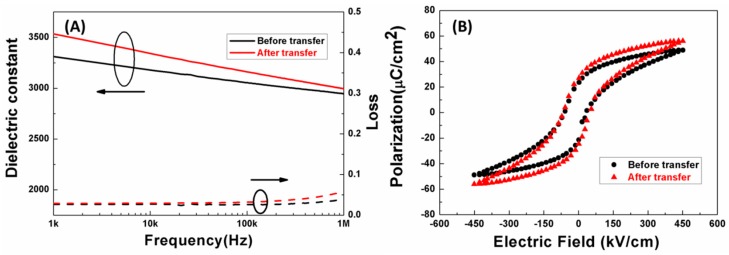
(**A**) Frequency dependent dielectric properties and (**B**) ferroelectric properties of the PMN-PT thick film before and after transfer.

As shown in Figure 4A, the dielectric constant curves exhibit a similar trend, decreasing with increasing frequency at a range from 1 kHz to 1 MHz. Clearly, PMN-PT thick film has a higher comparative dielectric constant after transfer. The main reason for this is reduced clamping [15]. Unlike the Silicon rigid substrate, E-solder 3022 belongs to a soft substrate and has less clamping effect on the thick film, which is beneficial for the domain movement. In addition, the dopant effect of alkali solution might also be beneficial for dielectric improvement. It is easy to see that the PMN-PT thick film on the Si substrate has an almost constant loss. However, the film’s loss slightly increases after transfer, and is still small enough for device application. In Figure 4B, the ferroelectric properties of PMN-PT film before and after transfer are presented. When the applied electric field is 450 kV/cm before transfer, the film’s remnant polarization (P_r_) is 23.1 C/cm^2^. After transfer, the P_r_ value of PMN-PT thick film increases to 26.8 C/cm^2^. This is likely due to easier domain motion under the condition of less clamping.

**Figure 5:**
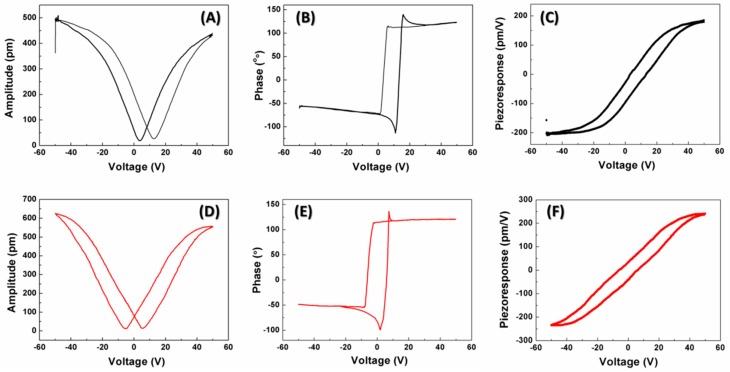
(**A**) Amplitude-voltage; (**B**) phase-voltage and (**C**) piezoelectric hysteresis loops of PMN-PT film before transfer; (**D**) amplitude-voltage; (**E**) phase-voltage and (**F**) piezoelectric hysteresis loop of PMN-PT film after transfer.

The piezoelectric performances of PMN-PT thick film before and after transfer were acquired using the AC modulation voltage of 2 V. Their amplitudes and phases dependent on the applied DC electric field are described in Figure 5A–D, respectively. The effective piezoelectric coefficient (d_33_,_f_) can be evaluated using the equation of d_33_,_f_ = A × cos ϕ/V. By substituting the values of A (amplitude), ϕ (phase) and V (AC modulation voltage) into it, the d_33_,_f_ of PMN-PT thick film on Silicon substrate was determined to be ~200 pm/V, while that of PMN-PT thick film on E-solder 3022 was calculated to be ~240 pm/V. This phenomenon indicates that the transfer process is beneficial for the piezoresponse enhancement of the PMN-PT thick film. According to this expression: d = 2QP_r_, where Q is electrostriction constant, the piezoelectric constant is directly proportional to the dielectric constant and remnant polarization. Thus, it is easy to see why the film’s piezoelectric property can be further improved after transfer.

## 4. Conclusions

A simple chemical transfer method was introduced to transfer the sol-gel derived PMN-PT thick film from Si substrate to conductive silver epoxy. The mechanism of this transfer has been well explained. The comparative investigation demonstrated that PMN-PT thick film could exhibit enhanced dielectric, ferroelectric and piezoelectric properties after transfer. Consequently, this transfer method is not only beneficial for performance improvement of the piezoelectric thick film, but also provides a simple way for high frequency transducer application, especially for ultrasound array fabrication. Further, this method could also prove suitable for other thick film’s transfer.
